# Development pattern of tracheal cartilage in human embryos

**DOI:** 10.1002/ca.23688

**Published:** 2020-10-08

**Authors:** M. Matthijs Fockens, Bernadette S. de Bakker, Roelof‐Jan Oostra, Frederik G. Dikkers

**Affiliations:** ^1^ Department of Otorhinolaryngology, Amsterdam University Medical Center location AMC University of Amsterdam Amsterdam The Netherlands; ^2^ Department of Medical Biology, Section Clinical Anatomy & Embryology, Amsterdam University Medical Center location AMC University of Amsterdam Amsterdam The Netherlands

**Keywords:** omplete l rings, trachea, tracheal bronchus, tracheal stenosis, tracheomalacia

## Abstract

**Introduction:**

Congenital tracheal anomalies are associated with high morbidity and mortality. The etiology of congenital tracheal anomalies is not well understood, but often attributed to malformed tracheal cartilage. The development of tracheal cartilage has not been described in detail. In this study, we aimed to investigate the development pattern and timing of normal tracheal cartilage to better understand the etiology of tracheal anomalies.

**Materials and methods:**

The development of tracheal cartilage was examined by studying the trachea in histological sections of 14 healthy human embryos from the Carnegie collection. Two specimens for Carnegie Stages 17–23 (42–60 days of embryological development) were studied.

**Results:**

At Carnegie Stages 17–19 (42–51 days), a continuous mesenchymal condensation was observed ventral to the tracheal lumen. At Stages 20 and 21 (51–54 days), this pre‐tracheal mesenchyme showed sites of increased condensation indicative of future tracheal rings. Furthermore, growth centers were identified both proximally and distally in the trachea. Characteristic horseshoe shaped tracheal rings were apparent at Carnegie Stages 22 and 23 (54–60 days).

**Conclusions:**

In human embryos, tracheal rings arise from growth centers in the ventral mesenchyme at approximately 51–54 days of embryological development. The observation of proximal and distal growth centers suggests a centripetal growth gradient, potentially contributing to occurrence of complete tracheal ring deformity (CTRD). Although this study shows new insights on tracheal cartilage development, the exact origin of congenital tracheal defects has yet to be elucidated.

## 1 INTRODUCTION

Congenital tracheal anomalies concern a spectrum of various rare developmental disorders often associated with malformed tracheal cartilage. These anomalies are seen primarily in neonates or infants, but are observed incidentally in older children or adults. Examples are tracheal stenosis with complete tracheal ring deformity (CTRD), tracheomalacia, tracheal bronchus, continuous cartilaginous sleeve, and tracheoesophageal fistula (Herrera et al., 2007). Although clinical presentation may range from asymptomatic to severe respiratory symptoms like biphasic stridor, dyspnea, and cyanosis, congenital tracheal anomalies are often associated with high morbidity and mortality (Chen & Holinger, 1994). These anomalies occur independently or in association with congenital birth defects like Down syndrome, Apert syndrome, Crouzon syndrome, or VACTERL association (Hamilton, Yaneza, Clement, & Kubba, 2016; Schweiger, Cohen, & Rutter, 2016; Zur, 2014). The cause of congenital tracheal anomalies or their relation with other congenital birth defects is not well understood.

The trachea is composed of 14–21 C‐shaped hyaline cartilage rings connected by annular ligaments (Kamel, Lau, & Stringer, 2009; Munguía‐Canales et al., 2011). The trachealis muscle forms the dorsal side of the trachea. Prior to embryonic development of the trachea, the primitive foregut is formed through cranial and lateral folding of the endodermal germ layer at approximately 3 weeks of embryonic development (Carnegie Stage 9, 19–21 days). The development of the tracheal lumen subsequently starts when the respiratory diverticulum is formed in the ventromedial part of the foregut at 26–30 days of development (Carnegie Stage 12). The respiratory diverticulum grows in a caudal direction, giving shape to the future trachea. At 28–32 days, the ventrally situated trachea separates from the dorsally situated esophagus and the respiratory diverticulum bifurcates into two primary lung buds (Carnegie Stage 13). The tracheal rings and lung mesenchyme derive from the mesodermal germ layer, as the endoderm only forms the epithelial lining of the respiratory tree. In embryological research, the mesenchymal precursors of tracheal rings have been described to some extent, but the development pattern and precise timing of tracheal cartilage remains unknown (Harjeet, Sahni, & Jit, 2004).

In this study, we aimed to investigate the embryonic development pattern and timing of normal tracheal cartilage as a first step to better understand the etiology of tracheal anomalies.

## 2 MATERIALS AND METHODS

Histological transversal sections of 14 healthy human embryos from the Carnegie collection were studied from the larynx to the main bronchi. The Carnegie collection is a human embryo collection assembled in the beginning of the 20th century by Franklin Mall of the Department of Embryology at the Carnegie Institution for Science (Mall & Meyer, 1921). This collection is considered a reference work for embryological research and its name is linked to the stages of embryonic development. The developmental age of the embryos used in this study ranged from 42 to 60 days, corresponding to Carnegie Stages 17–23. For each Carnegie stage, histological sections of two embryos were available for examination. The characteristics of these embryos (e.g., origin, year of acquisition, crown rump length, sex, fixation medium, staining, and number of slides per embryo) have been described elsewhere (de Bakker et al., 2016; de Bakker, de Bakker, Soerdjbalie‐Maikoe, & Dikkers, 2018). Carnegie collection specimen numbers used for this research are 6,521, 6,520, 6,524, 4,430, 2,114, 8,965, 462, 2,025, 7,254, 4,090, 895, and 950. These specimens were examined earlier during development of the interactive three‐dimensional (3D) atlas of the embryo (de Bakker et al., 2016). The 3D reconstructions of the trachea were made by manually selecting the thyroid cartilage, cricoid cartilage, tracheal rings, and bronchial rings in histological sections using Amira software (Thermo Fisher Scientific, Waltham, MA). If possible, the number of (future) tracheal rings was counted. Furthermore, the length of the trachea was calculated in the mid‐sagittal plane by measuring the distance between the cranial margin of the first tracheal ring (in Carnegie Stages 17–19, as no cricoid cartilage is discernable at these stages) or caudal border of the cricoid cartilage (in Carnegie stages 20 and up), and the carina.

## 3 RESULTS

Histological sections were of appropriate quality to accurately assess the trachea and potential tracheal rings in all embryos except for one (Carnegie Stage 23, specimen #9226). Furthermore, 3D reconstruction and measuring tracheal length was not accomplished in one embryo (Carnegie Stage 22, specimen #H983). In both cases, assessment was hampered by large section thickness and poor section quality. As the trachea runs in an oblique ventral‐superior to dorsal‐inferior course and the histological sections are in a purely transversal plane, accurate manual selection of each individual tracheal ring over the course of multiple sections was not possible. This pitfall complicated counting the individual number of tracheal rings in most embryos.

**FIGURE 1:**
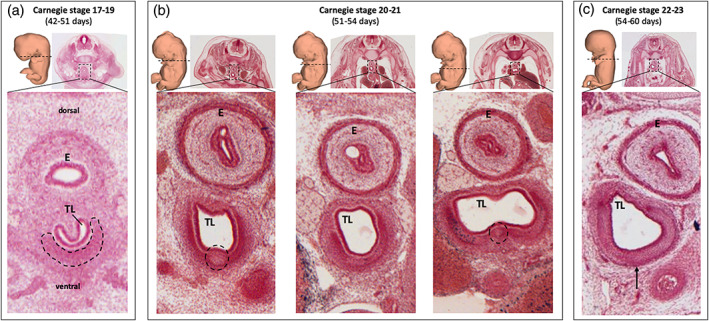
Histological sections of human embryos from the Carnegie collection. All presented sections are stained with aluminum cochineal and positioned in similar fashion, with the esophagus (E) located dorsal to the tracheal lumen (TL). (a) In Carnegie Stage 17–19, a horseshoe‐shaped mesenchymal pre‐cartilaginous condensation (dotted lines) is seen ventral to the tracheal lumen (specimen #6524). (b) In Carnegie Stage 20 and 21, cartilaginous nodules (dotted lines) are identified in the mesenchymal condensation proximally (left image) and distally (right image) in the trachea (specimen #462). In the middle of the trachea (middle image), mesenchymal precartilaginous condensation without cartilaginous nodules is observed. (c) In Carnegie Stage 22 and 23, horseshoe‐shaped cartilaginous tracheal rings can be seen (arrow; specimen #895)

In Carnegie Stages 17, 18, and 19 (42–51 days), average tracheal length was 1.2, 1.6, and 2.0 mm, respectively. A continuous mesenchymal condensation was seen ventral to the tracheal lumen (see Figure 1a). This mesenchymal condensation was C‐shaped, as no condensation was seen on the dorsal side. The uninterrupted pattern did not reveal the site of future tracheal rings.

In Carnegie Stages 20 and 21 (51–54 days), average tracheal length was 2.4 mm and 2.5 mm, respectively. The pattern of the ventral mesenchymal condensation was segmented over the entire length of the trachea, with an alternating increase and decrease of the intensity of the mesenchymal condensation. The sites of increased mesenchymal condensation (i.e., precartilaginous condensations) were considered to be indicative of future tracheal rings. Furthermore, rounded cartilaginous nodules (growth centers) could be identified ventromedially in these sites of pre‐cartilaginous condensations, ventral to the tracheal lumen (see Figure 1b). However, these cartilaginous nodules were seen only proximal and distal in the trachea. The middle part of the trachea did show an evidently segmented pattern of precartilaginous condensations, but no growth centers were identified. In one embryo of Carnegie Stage 20 (# 2025), approximately 14–16 sites of pre‐cartilaginous condensations were identified. Regardless of the presence or absence of cartilaginous nodules, the trachealis muscle was unaltered over the entire length of the trachea.

In Carnegie Stages 22 and 23 (54–60 days), tracheal length was 2.8 and 3.3 mm, respectively. The characteristic horseshoe‐shaped tracheal rings were visible and completely cartilaginous (see Figure 1c). In the embryo from Carnegie Stage 22 (specimen number 895), we identified 13 cartilaginous tracheal rings in the upper part of the trachea; in the lower part, it was not possible to differentiate individual tracheal rings. In the embryo from Carnegie Stage 23 (specimen number 950), 18 tracheal rings were identified.

## 4 DISCUSSION

**FIGURE 2:**
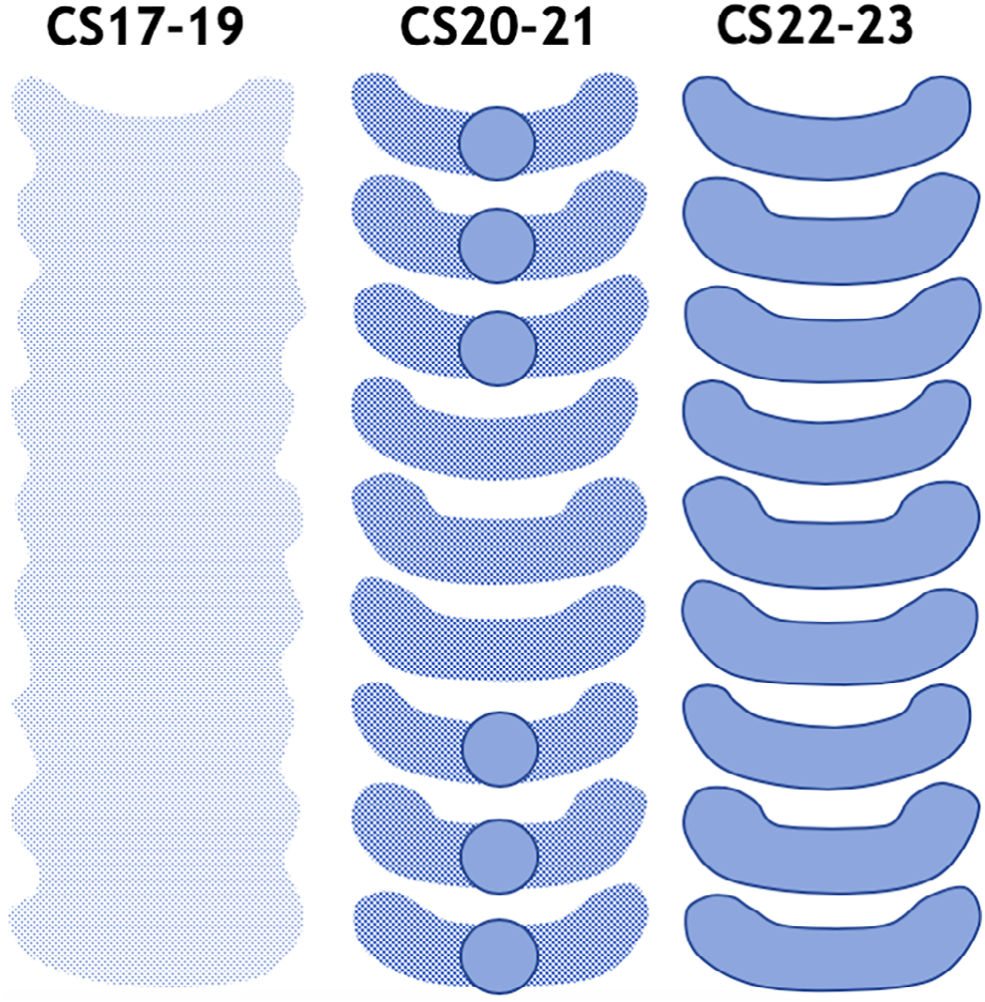
Schematic drawing of the supposed embryological development of tracheal rings. In Carnegie Stage 17–19, a continuous mesenchymal condensation is seen. Subsequently, in Carnegie Stage 20 and 21 sites of increased and decreased precartilaginous mesenchymal condensation are observed, with proximal and distal growth centers suggesting a centripetal growth gradient. Finally, horseshoe‐shaped cartilaginous tracheal rings are seen in Carnegie Stage 22 and 23

This is the first study to describe the human embryonic development of tracheal cartilage for every Carnegie stage (Figure 2). At Carnegie Stage 17–19, the continuous C‐shaped mesenchymal condensation ventral to the tracheal lumen is in accordance with the final shape of the horseshoe‐shaped tracheal rings. The sites of increased mesenchymal condensation at Carnegie Stage 20 and 21 are considered indicative of future tracheal rings. Interestingly, cartilaginous growth centers could only be discerned ventromedial in the pre‐cartilaginous condensations of the proximal and distal part of the trachea, while the middle part only shows pre‐cartilaginous condensations without cartilaginous nodules. From Carnegie Stage 22 onward, C‐shaped cartilage tracheal rings can be identified over the whole tracheal trajectory. To our knowledge, these findings have not been described in human embryos to this detail before.

In human embryos, (mesenchymal) condensation indicative of future tracheal cartilage was first described by Grosser (1912) and confirmed by Harjeet et al. (2004). This most recent publication stated tracheal cartilage development in four embryonic specimens with a crown rump lengths (CRL) of 11.5, 12.5, 22, and 30 mm (corresponding to Carnegie Stages 17, 18, 22, and 23, respectively). No growth centers were identified in that study. At 22 mm CRL, mesenchymal condensations were more pronounced cranially than caudally, suggesting a craniocaudal growth gradient. Interestingly, this is in contrast to our findings of proximal and distal growth centers suggesting a centripetal growth gradient.

In experimental animal studies, tracheal cartilage development has been described in accordance with our findings (Arooj Sher & Liu, 2016). In mice, tracheal cartilage development commences with mesenchymal cells situated ventrally to the trachea (Perl, Wert, Nagy, Lobe, & Whitsett, 2002). The ventrally located mesenchymal cells become committed to chondrocyte development and form cartilaginous nodules after proliferation, aggregation and condensation takes place. The cartilaginous nodules subsequently contain chondrocytes and eventually form cartilage. These animal studies describe an extensive spectrum of molecular mechanisms responsible for tracheal cartilage development, including transcriptional factor Sox9 and glycoprotein Sonic Hedgehog (Park et al., 2009). The molecular patterns in these animal studies were recently confirmed in a study on exome sequencing in CTRD patients, showing that CTRD is associated with gene mutations in genes encoding for cartilage formation, leading to absence of the trachealis muscle (Sinner et al., 2019).

Congenital tracheal anomalies concern a spectrum of various tracheal disorders. Some disorders can be explained by extra‐tracheal factors, such as congenital vascular abnormalities causing tracheal compression (e.g., pulmonary artery sling, double aortic arch, right aberrant subclavian artery) (Lodeweges et al., 2019). In this group of tracheal abnormalities, it is reasonable to consider the tracheal cartilage development as uninterrupted. In many other congenital tracheal anomalies, the abnormal development of tracheal cartilage itself is considered to be the cause of tracheal pathology.

Tracheomalacia, the most common congenital tracheal anomaly, is characterized by flattened C‐shaped tracheal rings causing collapse of the airway during inspiration. Although hypotheses on the origin range from insufficient strength of the tracheal rings to floppy collapse of the trachealis muscle, the exact cause is unknown. It occurs remarkably often in patients with tracheoesophageal fistula (37.4% in a recent study; Hseu, Recko, Jennings, & Nuss, 2015). Tracheoesophageal fistula is caused by incomplete foregut separation, leaving a connection between the trachea and esophagus. Experimental rodent studies have shown a role for Sonic Hedgehog among other transcription factors in development of tracheoesophageal fistula (Jacobs & Que, 2013). How tracheomalacia is linked to tracheoesophageal fistula has yet to be elucidated.

The most prevalent cause of congenital tracheal stenosis is complete tracheal ring deformity (CTRD) (Herrera et al., 2007). In CTRD, the cartilage is completely circular instead of horseshoe‐shaped and the trachealis muscle is absent. CTRD mainly occurs at the middle segment of the trachea and is accompanied by a variable length of tracheal stenosis. Sometimes CTRD is an incidental finding without causing symptoms of tracheal stenosis. CTRD is seen more often in patients with associated congenital malformations like Down syndrome, tracheal bronchus, or cardiovascular disorders like pulmonary artery sling (Bravo, Kaul, Rutter, & Elluru, 2006; Rutter, Willging, & Cotton, 2004). In mice, the pathogenesis of CTRD is attributed to disruption of a complex network of tracheal mesenchyme differentiation including Hedgehog and Wnt signaling pathways (Sinner et al., 2019). As it is generally assumed that tracheal cartilage develops over the entire length of the trachea at the same moment, the question why CTRD is regularly seen in the middle segment of the trachea remains unanswered. Based on our observation of proximal and distal growth centers, we hypothesize that growth centers forming tracheal cartilage first appear at the proximal and distal end of the trachea and subsequently appear in succession toward the middle of the trachea. By taking this longitudinal aspect into account when discussing the contributing factors in CTRD, we reckon that this centripetal growth gradient plays a role in the occurrence of CTRD in the middle segment of the trachea. Here, the combination of the disrupted signaling pathways and a centripetal growth gradient may lead to CTRD as unrestrained growth of the cartilaginous nodules may “fill the gap” of the absent trachealis muscle progenitor cells.

A tracheal bronchus (also called bronchus suis or pig bronchus, as it is normal in grazing animals including pigs, cattle, sheep, and giraffes) is defined as a displaced or supernumerary bronchus originating from the trachea (Frandson, Wilke, & Fails, 2009). The disorder is seen almost exclusively on the right side and occurs in 0.1–3% of the general population (Moreno et al., 2019). Theories on its development include absent regression of an additional lung bud and migration of bronchial mesenchyme into the trachea. As all segmental bronchi are completely formed at Carnegie Stage 15, it is unlikely that the development of tracheal bronchus is related to malformed tracheal cartilage.

Our study has some limitations. First, a small sample size allowed us to study tracheal cartilage in only 14 embryos spread over seven stages of development. Although our study clearly distinguishes three phases of tracheal cartilage development (i.e., continuous mesenchymal condensation at Carnegie Stage 17–19, segmented condensation with growth centers at Carnegie Stage 20 and 21, and tracheal rings at Carnegie Stage 22 and 23), we reckon that more detailed characteristics of tracheal cartilage development could be discerned if additional embryos would have been available. Second, not all embryos could be fully analyzed due to large section width and poor section quality. Third, counting the individual number of tracheal rings was complicated by the anatomical relationship between the oblique course of the trachea and the transversal plane of the histological sections.

Several questions remain to be studied. Are all tracheal rings developed simultaneously or in succession? If tracheal rings develop in succession, would the development occur in a centripetal, centrifugal, craniocaudal of caudocranial direction? If tracheal rings develop in a centripetal fashion, could unrestrained growth near the middle of the trachea be responsible for mid‐tracheal formation of CTRD and associated tracheal stenosis? Although this study shows interesting new insights on tracheal cartilage development, most aspects of the development of congenital tracheal anomalies remain unknown. As this study extensively studied tracheal cartilage development in the Carnegie collection, answering abovementioned questions will require future research on additional embryonic or fetal specimens.

In conclusion, tracheal cartilage development in human embryos takes place from Carnegie Stage 17–22 (42–58 days of development). The observation of proximal and distal growth centers suggests a centripetal growth gradient, potentially contributing to occurrence of CTRD. A lot of work on tracheal development remains to be performed to fully understand congenital tracheal anomalies.
